# A multiplex real-time PCR assay for routine diagnosis of bacterial vaginosis

**DOI:** 10.1007/s10096-015-2412-z

**Published:** 2015-07-05

**Authors:** J. G. Kusters, E. A. Reuland, S. Bouter, P. Koenig, J. W. Dorigo-Zetsma

**Affiliations:** Department of Medical Microbiology, University Medical Center Utrecht, G.04.614, P.O. Box 85500, 3508 GA Utrecht, The Netherlands; Medical Microbiology & Infection Control, VU University Medical Center, Amsterdam, Netherlands; Department of Medical Microbiology, Tergooi Hospital, Hilversum, Netherlands

## Abstract

A semi-quantitative multiplex PCR assay for the diagnosis of bacterial vaginosis (BV) was evaluated in a prospective study in a population of Dutch women with complaints of abnormal vaginal discharge. The PCR targets *Gardnerella vaginalis*, *Atopobium vaginae*, *Megasphaera* phylotype 1, *Lactobacillus crispatus* and *Lactobacillus iners*. Together with a short questionnaire, a vaginal swab for PCR and vaginal smear for microscopy were taken by their general practitioner or gynaecologist. Data from 151 women (median age 32) were available. Nugent Score (NS) was used to classify the samples and 83 samples were classified as normal (NS 0–3), 13 as intermediate (NS 4–6), and 55 as bacterial vaginosis (NS 7–10). In women with a NS of 7–10, PCR detected *Gardnerella vaginalis*, *Atopobium vaginae* and *Megasphaera* phylotype 1 in respectively, 96 %, 87 % and 60 %, whereas in women with a NS of 1–3 these species were detected in 27 %, 6 % and 2 % (*P* <0.001). A ratio of *Lactobacillus crispatus* over *Lactobacillus iners* of <1 (as calculated from the quantification cycle value (Cq)) was present in women with a NS of 7–10 in 66 % versus 33 % in women with a NS of 1–3 (*P* <0.001). The BV-PCR displayed a sensitivity of 92 % and specificity of 96 % with a positive predictive value of 94 % and a negative predictive value of 95 %. The Lactobacillus-index improved the correct classification of samples where only one of the other bacterial species was detected. Compared to the Nugent Score this multiplex qPCR offers a convenient tool for performing observer independent diagnosis of BV.

## Introduction

Abnormal vaginal discharge is the most common gynaecological condition for which a general practitioner is consulted. In the Netherlands there is an estimated incidence of 40–50 per 1,000 female patients per annum [1]. Trichomonas infections, *Candida albicans* infections, and bacterial vaginosis (BV) are among the top three causes of abnormal vaginal discharge. Moreover, BV has been associated with increased risk of adverse sequelae, such as preterm delivery, low birth weight, pelvic inflammatory disease and enhanced risk of acquiring STI [2].

Classically, BV is diagnosed according to the full criteria of Amsel et al. [3] or solely based on microscopy using either direct (wet mount) microscopy or microscopic examination of a Gram-stained vaginal specimen [4]. Routine culture of genital specimen reveals only part of the vaginal microbiota, and the presence of a culturable, well-known BV-associated species like *Gardnerella vaginalis* is relatively unspecific for diagnosis of BV [5].

Recently, cultivation-independent methods like PCR have revealed that the presence of newly discovered vaginal species such as *Atopobium vaginae*, unknown *Clostridiales* and *Megasphaera* spp., might be diagnostic of BV [6]. As the different vaginal species can be part of the normal vaginal microbiota, quantification can greatly improve the performance of these PCR-assays for diagnosis of BV [7–10]. Furthermore, the microscopic examination of vaginal specimen by the Nugent method is based on the relative abundance of *Lactobacillus* morphotypes (large Gram positive rods) compared to the abundance of bacterial morphotypes suggestive of BV, but it does not discriminate between the different *Lactobacillus* species. However, the ratio in which different *Lactobacillus* species appear in vaginal specimen can be predictive for normal or abnormal microflora: *L. crispatus* is found mainly in females with healthy vaginal microbiota, whereas *L. iners* is detected both in healthy and disturbed vaginal microbiota [9, 11–14]. The present study describes the development of a multiplex qPCR assay for the semi-quantitative detection of well-recognized BV-associated marker organisms and different *Lactobacillus* species and its evaluation in a clinical setting.

## Materials and methods

### Study population

The study was approved by the local ethical board of the investigating institute, Tergooi Hospital, Hilversum, the Netherlands, and written informed consent was obtained from participants.

A total of 159 women with self-reported complaints of abnormal vaginal discharge presenting to their general practitioner or gynaecologist between April 2009 and April 2010 were enrolled in the study. All enrolled were ≥18 years of age and had not received antibiotics or vaginal medications for at least 14 days prior to enrolment. The median age was 32 (range 18–62 years) and > 95 % were Dutch Caucasians. At the time of enrolment all participants completed a short questionnaire, and both a vaginal swab for PCR (Female multi-Collect Specimen Collection Kit; Abbott Molecular Inc., Des Plaines, IL, USA) and vaginal smear for microscopy were taken by the physician. Only if requested by the physician additional routine microbiological testing was performed. Evaluation could not be completed for eight of the patients, resulting in 151 patients for evaluation.

### PCR set-up

Based on the literature and preliminary studies five bacterial species were selected for a multiplex real time PCR set-up. These included three bacterial species (*Atopobium vaginae*, *Gardnerella vaginalis*, and *Megasphaera* phylotype 1) associated with bacterial vaginosis and two *Lactobacillus* species (*L. crispatus* and *L. iners*) detected both in the normal as well as in the disturbed vaginal microbiota [5–7, 11].

DNA was isolated from the swabs by automated DNA extraction on an Abbott M2000*sp* (Abbott, Illinois, USA) using the standard Chlamydia trachomatis/Neisseria gonorrhoeae DNA extraction protocol as advised by the manufacturer. The presence of the above-mentioned five bacterial species was established by an in-house semi quantitative real-time PCR. The specificity of the primer sets and probes (Table 1) was checked with PRIMER BLAST (http://www.ncbi.nlm.nih.gov/tools/primer-blast/) and all sets used were found to be highly specific for their intended targets. In addition we tested our multiplex qPCR with a large collection of strains both for each of the bacterial targets as well as with related bacterial species, and only the intended targets would show up as positive (data not shown). The qPCR reactions (see Table 1 for details) were performed as two multiplex reactions in a Corbett RotorGene 6000 PCR machine (Qiagen, Hilden, Germany) using Qiagen QuantiTect Multiplex Taq polymerase and corresponding buffers as advised by the manufacturer. Primer concentration was 0.5 μM for all forward and reverse primers and 0.25 μM for all probes. Reactions were performed in a final volume of 20 μl containing 5 μl DNA eluate. PCR conditions consisted of a heat-activation of the Taq polymerase of 95 °C for 10 minutes followed by 40 cycles of amplification. Each amplification cycle consisted of a denaturation step at 95 °C for 15 seconds and an annealing/amplification step at 55 °C for one minute. In all qPCR runs positive controls for all targets as well as negative controls (H_2_O instead of purified sample DNA) were always included. In addition multiplex I (See Table 1) included a primer/probe set for detection of human β-globulin DNA to check for adequate sample collection and preservation and isolation of DNA of sufficient quality. The qPCR results of clinical samples were only considered valid if the β-globulin PCR resulted in a quantification cycle (Cq) value ≤35 and second derivatives of the amplification plots were calculated to determine the fractional qPCR cycle value. Bacterial PCRs were scored as positive if a clear S-curve was observed in the respective amplification plot.

**Table 1:** Primer sets and amplicon length of the two multiplex PCRs

PCR target	Primer name	Oligo composition (5′–3′)^a^	Amplicon size
Multiplex 1			
β-globulin^b^	Forward	GAAGAGCCAAGGACAGGTAC	268 bp
	Probe	[Cy5]TCTGCCGTTACTGCCCTGT	
	Reverse	CAACTTCATCCACGTTCACC	
*L. iners*	Forward	AGTCTGCCTTGAAGATCGG	166 bp
	Probe	[FAM]CCAAGAGATCGGGATAACACCT	
	Reverse	CTTTTAAACAGTTGATAGGCATCATC	
*L. crispatus*	Forward	AACTAACAGATTTACTTCGGTAATGA	145 bp
	Probe	[ROX]CCCATAGTCTGGGATACCACTT	
	Reverse	AGCTGATCATGCGATCTGC	
Multiplex 2			
*A. vaginae*	Forward	TAGGTCAGGAGTTAAATCTG	155 bp
	Probe	[HEX]CTACCAGACTCAAGCCTGCC	
	Reverse	TCATGGCCCAGAAGACCGCC	
*G. vaginalis*	Forward	GCGGGCTAGAGTGCA	206 bp
	Probe	[ROX]CTTCTCAGCGTCAGTAACAGC	
	Reverse	ACCCGTGGAATGGGCC	
*Megasphaera* *phylotype 1*	Forward	GATGCCAACAGTATCCGTCCG	208 bp
	Probe	[FAM]ACAGACTTACCGAACCGCCT	
	Reverse	CCTCTCCGACACTCAAGTTCGA	

^a^Primers and probes were obtained from TIB MOLBIOL GmbH, Berlin, Germany. Cyanine 5 (Cy5), Fluorescein (FAM), X-Rhodamin (ROX) and Hexachlorfluorescein (HEX) were used as the 5′-coupled reporter fluorophores of the hydrolysis probes used in the multiplex PCR reaction, and the 3′-coupled Black Hole Quencers (BHQ1 and BHQ2) as quenchers

^b^β-globulin PCR was used as a sample and DNA/PCR quality control. See the “Materials and methods” section for explanation

### Lactobacillus index

For the presence of *Lactobacillus* species an index was calculated: in case of ≥5 Cq value difference between the two *Lactobacillus* species and a higher load of *L. iners* detected, the ‘Lactobacillus-index’ (L-index) is <1 (indicating a shift to disturbed vaginal microbiota); in case a higher load of *L. crispatus* was detected, the L-index is >1 (indicating healthy vaginal microbiota); in case both *Lactobacillus* species were detected in the same amounts, or both were absent, the L-index = 1 (undetermined vaginal microbiota).

### Vaginal smears

Vaginal smears were Gram-stained and scored using Nugent Score (NS). Based on their NS patients were subdivided in three groups: normal vaginal microbiota (NS 0–3), intermediate vaginal microbiota (NS 4–6) and bacterial vaginosis (NS 7–10). Slides were scored independently by two experienced technicians. In case of discordant results (*n* = 43/151; 28 %), slides were re-examined by the same technicians, unaware of their previous score. For vaginal smears remaining with discordant results after this second round (*n* = 8), the microscopists sat together and came to consensus after a final round of revisions.

### Statistical analysis

The Cq values for both *Lactobacillus* species were determined and when appropriate the L-index (ratio of *L. crispatus* over *L. iners*) was calculated from these Cq values as described above. Statistical analyses were performed with SPSS, version 20.0. Proportions were compared using Pearson's X^2^, Fisher’s exact test and Mann–Whitney U test where appropriate. Scatter plots were prepared using Prism V6.0 for Windows (GraphPad Software Inc., San Diego, CA).

## Results

### Characteristics of women enrolled in the study

For 151 women, a vaginal swab for PCR, a vaginal smear for microscopy, informed consent and a completed questionnaire were received. For 149 of them, additional microbiological testing, i.e. routine culture of the genital specimen (*n* = 149) and molecular diagnostics for *Chlamydia trachomatis* and *Neisseria gonorrhoeae* (*n* = 114), *Trichomonas vaginalis* or herpes simplex virus (HSV), was performed.

Of the 151 women, 83 (55 %) had normal vaginal microbiota, 13 (9 %) intermediate vaginal microbiota and 55 (36 %) had bacterial vaginosis according to NS. The clinical and laboratory characteristics of this population are shown in Table 2. Sexually transmitted infections (STIs) among these 151 patients were uncommon; one woman had *C. trachomatis*, one had *N. gonorrhoeae*, three had *T. vaginalis* and three had HSV infection. Further pathogens detected were: *Candida* species, mostly *C. albicans* (in 39 women), *Haemophilus influenzae* (in three), and Group A β-hemolytic *Streptococcus* (in two). Seventy-four women used (oral) contraceptives (OC), eight were pregnant and 115 had visited their physician for abnormal vaginal discharge in the preceding two years.

**Table 2:** Clinical and laboratory findings in 151 females with abnormal vaginal discharge

Patient sample characteristics	Nugent score 0–3 (*n* = 83)	Nugent score 4–6 (*n* = 13)	Nugent score 7–10 (*n* = 55)	*P-value*
Age (median) when obtaining sample^a^	31.7 (25–40.4)	32.3 (22.4–41.5)	33.1 (24.8–41.3)	0.981^c^
Patients using contraceptives^b^ (data based on 149/151 patients)	39 (50 %) 35 OC, 4 IUD/other	6 (46 %) 5 OC, 1 IUD/other	29 (58 %) 19 OC, 10 IUD/other	0.376 0.442 0.009
Vaginal discharge				
Patients^b^ with known prior events of abnormal vaginal discharge (data based on 146/151 pts)	50 (63 %)	11 (85 %)	32 (60 %)	0.805
Known non-BV cause of vaginal discharge				
-*Candida sp.*	21 (25 %)	5 (39 %)	13 (24 %)	0.855
-Other	7 (8 %)	1 (8 %)	2 (4 %)	0.483
Presence of Gardnerella/Atopobium/Megasphaera				
*Gardnerella* (total no. of positive samples)	22 (27 %)	8 (62 %)	53 (96 %)	<0.001
No. of samples with Cq ≤20	6 (7 %)	3 (23 %)	30 (55 %)	<0.001
*Atopobium* (total no. of positive samples)	5 (6 %)	4 (31 %)	48 (87 %)	<0.001
No. of samples with Cq ≤25	1 (1 %)	3 (23 %)	29 (53 %)	<0.001
*Megasphaera* (total no. of positive samples)	2 (2 %)	2 (15 %)	33 (60 %)	<0.001
No. of samples with Cq ≤25	0	1 (8 %)	21 (38 %)	<0.001
Lactobacillus index < 1	27 (33 %)	6 (46 %)	36 (66 %)	<0.001

*OC* oral contraceptives, *IUD* intra uterine device

*P*-values are a comparison between women with NS 0–3 and those with NS 7–10

^a^Variables are denoted as median (interquartile range)

^b^Data are only available for the indicated subset of patient samples

^c^Group differences were tested with the Mann–Whitney U test

### PCR-based detection of *G. vaginalis*, *A. vaginae*, *Megasphaera*, *L. crispatus* and *L. iners*

**Fig. 1:**
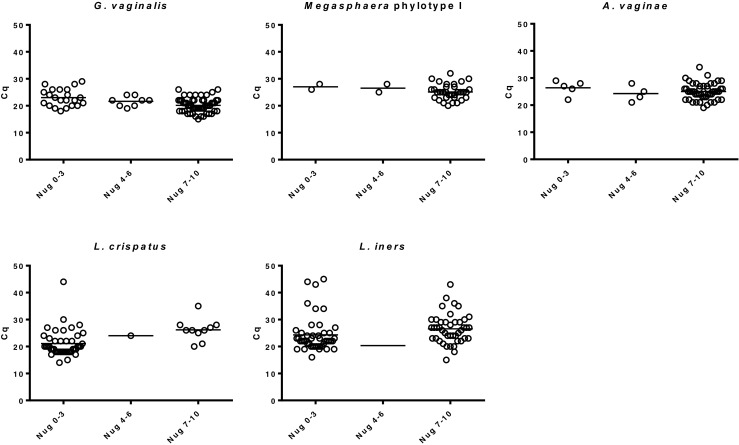
Relation between Cq values and Nugent scores. Each panel represents the Cq values obtained in the multiplex qPCR for each of the five bacterial species tested. *Circles* represent Nugent score and Cq values of a single patient grouped per patient category. Nugent scores 0–3 (indicating normal), 4–6 (intermediate) and 7–10 (indicative of bacterial vaginosis) are presented on the X-axis, and Cq scores on the Y-axis. This figure represents the results of all 151 patient samples analysed in this study (see also Table 2)

In women with BV (NS 7–10), *G. vaginalis*, *A. vaginae*, and *Megasphaera* phylotype 1 were present in 96 %, 87 % and 60 % of the vaginal specimens, respectively, whereas in women with normal vaginal microbiota (NS 0–3) these bacteria were detected in, respectively, 27 %, 6 % and 2 % (*P* <0.001). To establish the quantitative relationship between bacterial load and NS, bacterial loads were plotted against the NS in scatter plots and the median Cq value at which the bacterium was first detected among all samples was calculated (Fig. 1). Median Cq values for *G. vaginalis*, *A. vaginae*, and* Megasphaera* phylotype 1 were 20, 25 and 25, respectively. Higher loads of *G. vaginalis* (Cq ≤ 20), of *A. vaginae* (Cq ≤ 25), and of *Megasphaera* (Cq ≤ 25) were significantly more common in women with BV than in women with normal vaginal microbiota (*P* <0.001) (Table 2). An L-index <1 (shift to disturbed vaginal microbiota) was present in women with BV in 66 %, compared to 33 % in women with normal vaginal microbiota according to NS (0–3) (*P* <0.001).

The proportion of women with *G. vaginalis*, *A. vaginae*, *Megasphaera* phylotype1 detected by PCR and the calculated L-index, stratified according to NS, are shown in Table 2.

### BV-PCR assay validation

Subsequently, the multiplex qPCR assay for molecular diagnosis of BV (BV-PCR) was compared with the NS as reference method for the diagnosis of BV. BV-PCR was scored positive in case ≥ 2 BV-associated bacteria were detected, regardless of the L-index; BV-PCR was scored negative in case no BV-associated bacterium was detected, regardless of the L-index or one BV-associated bacterium was detected and L-index ≥1; BV-PCR was scored indeterminate in case one BV-associated bacterium was detected and L-index <1. Thus 55 samples were scored BV-PCR positive, 86 BV-PCR negative and ten BV-PCR indeterminate. A correlation between BV-PCR scores (either positive, indeterminate, or negative) and the corresponding Nugent scores (bacterial vaginosis, intermediate, or normal) of the samples was observed with negative PCR samples almost always having a normal NS and vice versa (not shown). Sensitivity and specificity, PPV and NPV of the BV-PCR was calculated using all samples (*n* = 131) for which both NS and BV-PCR results were conclusive (either positive or negative) for diagnosis of BV. The BV-PCR was highly sensitive: 92 % and specific 96 % for diagnosis of BV, with a PPV of 94 % and NPV of 95 % for predicting BV.

## Discussion

The present study describes the development and clinical validation of a multiplex qPCR for the molecular diagnosis of BV. The vaginal microbiota is composed of numerous bacterial species, and recently researchers sought for correlations between the presence or absence of these species and the clinical condition of BV [5–10, 12, 13, 15]. Building on these previous reports, we selected three BV-marker organisms to include in our multiplex PCR. In addition, two *Lactobacillus* spp. were incorporated in the qPCR as targets. *Lactobacillus* spp. are believed to play an important role in maintaining a healthy vaginal environment [16], and where *L. crispatus* has been found mainly in healthy vaginal microbiota, *L. iners* is prevalent in both healthy and disturbed vaginal microbiota. As the vaginal microbial environment is dynamic, and transient phases between healthy microbiota and BV occur, we sought to incorporate these two *Lactobacillus* spp. in a quantitative relation to each other, in order to enable classification of transient phases in vaginal specimens. Besides, the relative quantitation of Gram positive rods, morphology consistent with *Lactobacillus* spp., constitutes a significant component in the frequently performed Gram-stain for diagnosing BV and therefore it seemed reasonable to incorporate *Lactobacillus* species in a molecular test for BV.

We applied the multiplex qPCR (BV-PCR) in a prospective study among females with complaints of abnormal vaginal discharge. In contrast to previous studies for molecular diagnostics of BV [5, 8, 15], participants were not recruited from an STD clinic or among pregnant women. Indeed, having an STI, engagement in same sex behaviour or the amenorrheic state in pregnancy can influence vaginal microbiota [17, 18] and therefore clinical validation of a diagnostic test in samples from study participants recruited through general practitioners and gynaecologists like in our study seems more appropriate. The additional information obtained from the participants revealed no significant differences between the groups with NS 0–3 and NS 7–10 with regard to either age, contraceptive use, previous reported abnormal vaginal discharge, or other putative causes of vaginal discharge being present.

In the clinical validation of our assay, we observed that the presence of the combination of BV-marker organisms results in a better association with BV than the individual scores of either of these bacteria. Adding the Lactobacillus-index, which on its own was not predictive for BV, allowed classification of those samples in which only one BV-marker organism was detected. Compared to the reference method (Nugent Score on vaginal specimen), our BV-PCR showed a high sensitivity (92 %) and specificity (96 %) for diagnosis of BV, confirming the potential diagnostic value of molecular diagnosis of BV. Also in this study we found initially 28 % of the vaginal smears discrepant in Nugent score as classified by two experienced technicians, whereas PCR results were highly reproducible and technician independent (data not shown). Furthermore, in contrast to the Nugent scoring system, in which abundant numbers of *Lactobacilli* (Gram positive rods) are scored as healthy vaginal microbiota, discrimination and quantification of the two *Lactobacillus* species in the qPCR generates information on a shift of *Lactobacillus* populations in the vagina. Recently, both in studies detecting *Lactobacillus* by qPCRs [5, 9, 18] or by broad range 16 s rRNA PCRs [12, 19–21], *L. iners* was the most prevalent species in the vagina, but in contrast to *L. crispatus* it is common and abundant in vaginal microbiotas with high concentrations of BV-marker organisms [11, 12]. Also in our study we found significantly higher loads of *L. iners* (i.e. L-index <1) in women with BV, although in nearly half of the women without BV a higher load of *L. iners* compared to *L. crispatus* (i.e. L-index <1) was detected too. Whether this condition reflects a transient phase from predominance of healthy to disturbed vaginal microbiota requires follow-up studies within the individual female.

Although in previous clinical studies high loads of *G. vaginalis* and *A. vaginae* have been shown to correlate with a diagnosis of BV [8, 15], recently substantial variability in *G. vaginalis* loads with the menstrual cycle has been demonstrated [22]. We defined BV-PCR positivity relying on the composition of vaginal microbiota rather than the individual loads of the targeted BV-marker organisms. Additionally we used the load of different *Lactobacillus* species in indicating a shift in vaginal microbiota. Thus we reached an excellent sensitivity and specificity for the BV-PCR assay. As with Nugent based scoring also the BV-PCR test resulted in inconclusive results for a small number of patients (respectively, 9 % and 7 %). From preliminary studies (data not shown), apart from *G. vaginalis* and *A. vaginae*, *Megasphaera* phylotype 1 was the most promising in molecular based diagnosis of bacterial vaginosis. It is possible that by adding other relevant species to a multiplex PCR the molecular diagnosis of BV can be even more refined compared to our BV-PCR assay.

In conclusion, compared to the reference Nugent score in females suffering from abnormal vaginal discharge, the described multiplex qPCR was found to be a reliable tool for diagnosis of BV. The main advantage of the BV-PCR is that it can be performed in a reproducible and standardized fashion that does not depend on subjective interpretation of the examiner as is the case with the frequently used scorings such as classical wet mount microscopy or Nugent score [23]. In addition this qPCR based method can easily be incorporated in a fully automated PCR workflow as the Qiagen QIAsymphony and the Roche FLOW, thus expanding the existing portfolio of high through-put routine diagnostic tests of a modern clinical microbiological laboratory. Further development of this assay might ultimately allow to translate it into a cheap and reliable point-of-care test. Since BV is a common, often transient but recurrent clinical condition, this diagnostic BV-PCR can be applied in follow-up diagnostics within the same female. As such it is suitable to elucidate the role of the different targeted species in the pathogenesis of bacterial vaginosis.
